# A brief review and guidance on the spatiotemporal sampling designs for disease vector surveillance

**DOI:** 10.1016/j.crpvbd.2024.100208

**Published:** 2024-08-15

**Authors:** Abdollah Jalilian, Jorge Mateu, Luigi Sedda

**Affiliations:** a Lancaster Ecology and Epidemiology Group, Lancaster University, UK; b Department of Mathematics, Universitat Jaume I, Spain

**Keywords:** Optimal spatiotemporal sampling, Design-based and model-based sampling designs, Surveillance, Vector-borne diseases

## Abstract

Obtaining a representative sample of disease vectors (mosquitoes, flies, ticks, etc.) is essential for researchers to draw meaningful conclusions about the entire vector population in a target study area and during a specific study period. To achieve this, a carefully chosen surveillance design is required to ensure that the sample captures essential spatial and temporal variations in the target vector population(s) and/or that the study results can be generalized to the entire population. Designed-based and model-based spatiotemporal sampling (or in our context surveillance) designs can be used to maximize information gain within given resource constraints. In this paper, we aim to offer a concise overview of common spatiotemporal field sampling designs, their advantages and disadvantages and their practical applications in the context of surveillance and management of vector-borne diseases. At the end of the article, we offer guidance to help vector-borne disease surveillance planners design effective spatiotemporal surveillance interventions.

## Highlights

• An overview of the main approaches and designs for spatio-temporal surveillance is provided.
• Non-probabilistic preferential sampling can result in biased estimations of the mosquito parameters of interest.
• When little information is available, a mixed random and grid survey is an optimal solution.
• When previous information is available, adaptive designs can provide better gains towards a target.

## Graphical abstract

**Figure:**
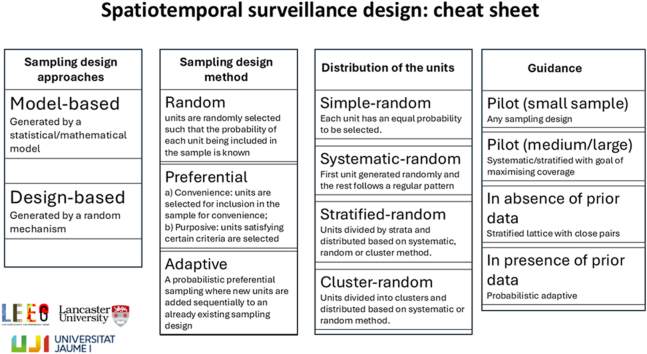

## 1 Introduction

Vector-borne diseases are mostly transmitted by arthropods, such as mosquitoes, ticks, fleas, lice, and sandflies, and are the cause of 700,000 deaths annually and millions of infections worldwide. The burden of these diseases is the highest in tropical and subtropical areas (WHO, 2020). Vector-borne diseases originate from a complex interaction between vectors, animal hosts (if required), and environmental, climatic, geographical, and socio-economic factors, including temperature, humidity, vegetation, pollution, latitude, elevation, urbanization, and economic development (Reisen, 2010; Parham et al., 2015). Over the past decades, researchers and public health managers have employed various spatial and temporal statistical methods and used satellite imagery and other remote sensing data to understand and quantify the impact of these factors on the spatiotemporal variability of vector-borne diseases (Kitron et al., 1998; Palaniyandi, 2012).

These technological advancements combined with the adoption of open data policies and data becoming more accessible even at fine spatial resolution (Palaniyandi, 2012) had sparked the use of ‘statistically informed’ spatial sampling for vectors that for the scope of this review, we use as synonym for spatial vector surveillance. Statistically informed vector sampling has been applied to initial (Sedda et al., 2019) or adaptive (Monteiro et al., 2024) spatiotemporal surveillance designs, especially in low- and middle-income countries where economic resources are usually scarce and therefore surveillance or control optimization is a necessary task (Reisen, 2010). By carefully choosing a spatiotemporal sampling design, researchers can draw meaningful conclusions about the spatial distribution, species diversity, behavior, temporal trends, seasonal variations, and other ecological or entomological characteristics of the target vector population or populations in the study area (Parham et al., 2015).

The process of sampling a population or metapopulation of disease vectors typically involves two steps. First, an appropriate spatiotemporal sampling design is used to select specific sampling sites (locations or regions) with their corresponding sampling times and frequency for investigation. Following this, in the selected sampling sites and at the chosen sampling times, a suitable vector collection method is applied to obtain a representative sample within the study area and during the study period (Focks & UNDP/World Bank/WHO Special Programme for Research and Training in Tropical Diseases, 2004; Springer et al., 2016). Common vector collection methods include traps (e.g. light, gravid, sticky, and pheromone traps), human landing catches (mainly for mosquitoes), aspirators (used for most of the vectors), netting (for airborne or vegetation-dwelling vectors), and larval surveys (particularly for water-breeding vectors like mosquitoes) (Silver, 2007). It is important to point out that effectiveness differs between these collection methods, and readers are referred to the vast literature on this topic, e.g. Degener et al. (2014).

The spatial and temporal scales of any spatiotemporal sampling design depend on the size and scope of surveillance effort and it is bound by logistical and financial constraints (Springer et al., 2016). Consequently, the use of appropriate spatiotemporal sampling designs becomes pivotal for extracting maximum information within these resource constraints (Sedda et al., 2019). In this paper, we provide a brief overview of common spatiotemporal sampling methods and their applications in the surveillance and control of vector-borne diseases, present their advantages and disadvantages, and provide general guidance.

## 2 Spatiotemporal sampling

In the study of a disease vector, particularly in entomology, the attention is usually concentrated to a specific variable related to its population, such as vector density, distribution, bionomics, insecticide resistance, and infection rate (Bowman et al., 2014). This investigation involves examining variations of one or more of these study variables, but for simplicity here we consider a single study variable as Y, defined within a geographical study area and over a study period.

The study area is typically a bounded spatial domain (confined), denoted as D. It consists of distinct spatial units, which represent individual locations (villages or other locations of interest such as provinces, districts, or regions) distributed throughout the study area. On the other hand, the study period refers to the specific timeframe during which the investigation takes place. It can be represented by the temporal interval $T=\left[t_{\min},t_{\max}\right]$, where $t_{\min}$ and $t_{\max}$ denote the starting and ending time points of the investigation, respectively. Any spatial unit, denoted as u (represented by location attributes such as latitude and longitude for each point or vertex of a polygon), is positioned within the study area D, and any temporal unit, denoted as t, represents a specific time instance during the study period T.

Apart from investigating variations in the study variable Y(u,t) across spatial units u∈D and temporal units t∈T, epidemiologists and medical entomologists are interested in exploring the potential impact of environmental variables (for example temperature, humidity, rainfall, elevation, and vegetation) or other variables, on Y(u,t).

In a discrete spatial domain, the study area D is divided into spatial units, i.e. $D=\left\{u_{1},\ldots ,u_{N}\right\}$, and the study variable Y(u,t) is observable in all or some of these defined spatial units. Examples of such discrete spatial units for vectors such as mosquitoes include predetermined breeding sites (rice fields, irrigation canals, ponds, marshes, water storages, sandy or moist soil, forests, grasslands, shrubs), resting sites (vegetation, animal shelters, and human dwellings), and feeding sites (e.g. human settlements and livestock buildings) (Silver, 2007).

**Fig. 1:**
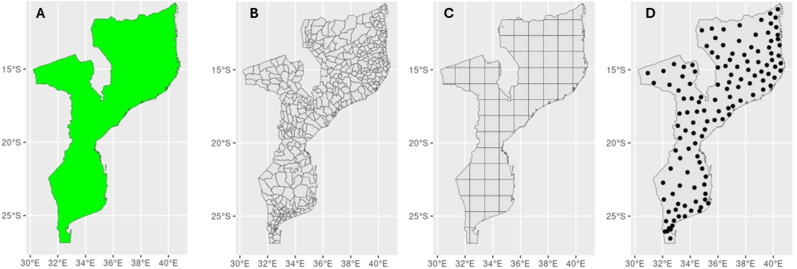
Examples of a continuous spatial domain (**A**) where any point inside the boundary is potentially a spatial unit; and discrete spatial domains with districts (**B**), grid cells (**C**), and settlements (**D**) as spatial units.

In contrast, within a continuous spatial domain, every location u across the entire study area D is considered a potential spatial unit where the study variable Y(u,t) can be measured. However, this is unlikely to be the case in most studies, due to practical (economic and/or logistical) and computational considerations, and therefore continuous spatial domains are often discretized. This discretization process involves techniques such as choosing central points within a fine regular grid or forming tessellated cells for more efficient computations. The discretized spatial domain is, again, a finite set $D=\left\{u_{1},\ldots ,u_{N}\right\}$, where N is relatively large. The spatial units $u_{1},\ldots ,u_{N}$ represent the defined points, polygons, cells or segments within the domain D. Fig. 1 illustrates examples of discretizing a continuous spatial domain (Fig. 1A) into spatial units using administrative polygons (Fig. 1B), grid cells (Fig. 1C) or settlements (Fig. 1D).

Nevertheless, the choice between studying disease vectors at predefined locations within the study area (discrete spatial domain) or conducting a comprehensive study across the entire study area (continuous spatial domain) depends on the research objectives, resource availability, accessibility, and logistical constraints (Gruijter et al., 2006). In practice, if the primary interest of the study is solely in determining the mosquito prevalence rate per household within the country, then every household across the country is a distinct well-defined spatial unit and the spatial domain is classified as discrete. If the research objective involves creating a comprehensive map depicting the density of mosquitoes throughout the entire country, since disease vectors can potentially exist everywhere, any location within the countryʼs boundaries becomes a potential spatial unit and the spatial domain is continuous.

Similarly for space, the study period T is often partitioned or aggregated into appropriate temporal units which can be represented as $T=\left\{t_{1},\ldots ,t_{m}\right\}$, where $t_{\min}\leq t_{1}<\cdots <t_{m}\leq t_{\max}$. The selection of the number of units (scale, e.g. yearly, monthly, weekly, daily, or hourly) and duration is usually based on the behavior, seasonality, and dynamics of the vector population and again by logistical constraints.

For instance, malaria mosquitoes typically have a relatively brief life span, lasting approximately 2–3 weeks. They also exhibit a clear seasonal pattern, with increased abundance and activity levels commonly observed during the rainy season and mainly at night (Bousema and Drakeley, 2017). As a result, the standard practice involves conducting mosquito sampling on a weekly or bi-weekly basis, primarily during the nighttime hours and throughout the malaria season to understand the temporal dynamics of *Anopheles* mosquitoes and relate them to malaria outbreaks (Sedda et al., 2022).

## 3 Design-based and model-based spatiotemporal sampling approaches

Once the maximum number of spatial units $D=\left\{u_{1},\ldots ,u_{N}\right\}$ and the temporal units $T=\left\{t_{1},\ldots ,t_{m}\right\}$ are clearly defined, a spatiotemporal sampling design with sampling effort *n* (0 < *n* < *N* × *m*) is represented as $S=\left\{\left(u_{1},t_{1}\right),\ldots ,\left(u_{n},t_{n}\right)\right\}$, with sampling locations $u_{1},\ldots ,u_{n}$ and sampling times $t_{1},\ldots ,t_{n}$.

Having a sampling design is the cornerstone for any statistical inference method to draw conclusions about the entire vector population based on the selected sample, and enhances the precision and accuracy of the findings. However, the critical decision regarding the most appropriate sampling design depends on the selection between two distinct approaches to statistical inference (Gruijter et al., 2006).

• Model-based approach where a statistical model is employed to describe the underlying process governing the variations of the study variable. This approach targets both the expected values of the investigated process and its stochasticity (i.e. variation around the average values of the process under study at any given location in space and time).
• Model-free or design-based approach where a well-defined random mechanism with known probabilities is employed to select a sample from spatial and temporal units. This approach targets the expected values of the investigated process but not its stochasticity.

Table 1 summarizes the basic assumptions, advantages, and disadvantages of both model-based and design-based approaches. In general, design-based approaches should be applied when information about the studied process is not available and little is known about the area of investigation (Mateu and Müller, 2013).

**Table 1:** Comparison of assumptions, advantages, and disadvantages of model-based and design-based approaches for drawing conclusions about the entire population from a selected sample.

	Model-based approach	Design-based approach
Assumption	Given the sampling design S, the study variable Y(u,t) is generated by a statistical model.	Given the variable of study Y(u,t), the sampling design S is generated by a random mechanism.
Advantages	Can provide more precise estimates and predictions if the model and distribution assumptions for the study variable are accurate.	No assumptions about the underlying distribution for the study variable are needed.
Disadvantages	Requires strong assumptions about the data-generating process and is sensitive to model misspecification.	Requires larger sample sizes to achieve the same level of precision of a model-based approach.

A model-based approach specific for mapping and prediction of the study variable Y(u,t) across all spatial and temporal units using observations at a finite number of locations and times is model-based geostatistics (Diggle et al., 2013), which requires the definition of the trend or covariates (or independent variables, or fixed effects), the function of the spatiotemporal covariance structure (e.g. Matern covariance function), and a suitable distribution for the generalized linear model (normal, binomial, Poisson, etc.). The parameters in the model are inferred for describing variation of the study variable and the effect of observed and unobserved relevant factors in this variation. If model assumptions are correct and the set of parameters is known, then values of the study variable at all spatial and temporal units can be predicted using the model and values of the study variable at the sampling points. However, in practice the parameters are not known in advance, and hence they need to be estimated based on values of the study variable at sampling points (statistical inference).

The entire process of making statistical inference about the vector population within a model-based approach can be summarized in the following steps.

1. Specify an appropriate parametric model to describe variations of the study variable and the contribution of both observed and unobserved relevant factors.
2. Select a suitable sampling design to determine the number and points (locations and times) of samplings.
3. Estimate the model parameters based on values of the study variable at sampling points.
4. Predict values of the study variable at all spatial and temporal units based on the specified model and values of the study variable at sampling points.

**Fig. 2:**
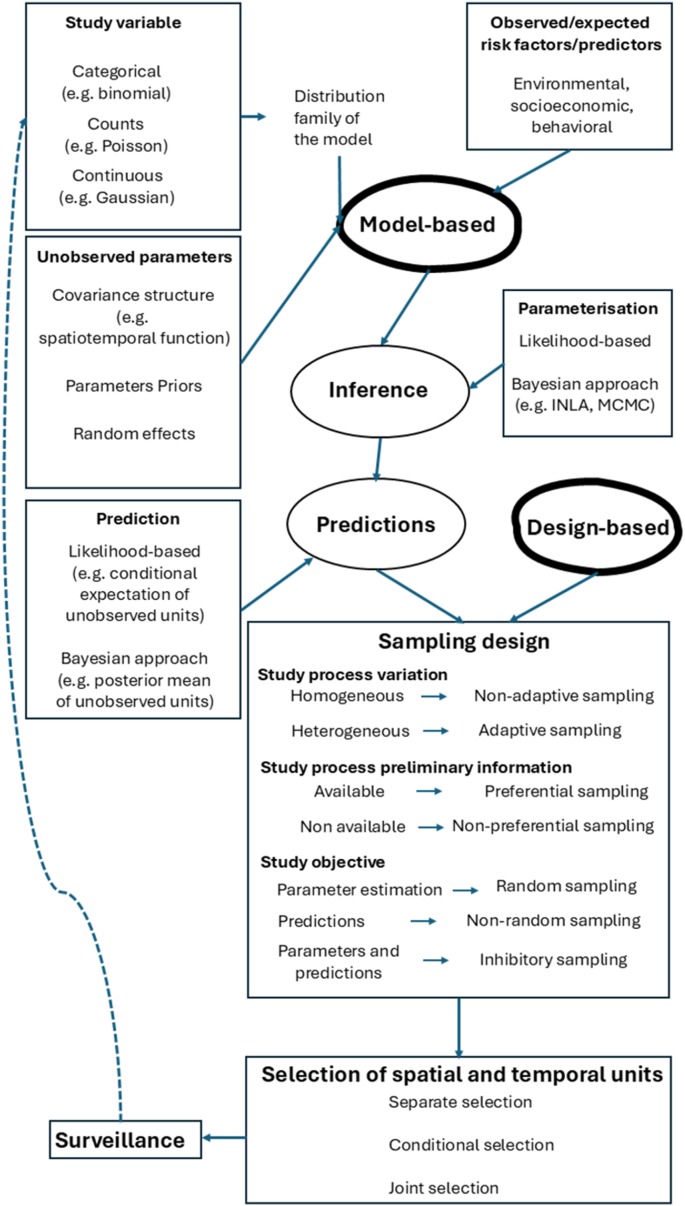
A graphical description of a spatiotemporal sampling design for estimation and prediction of the study variable.

Figure 2 provides a detailed graphical description of the model-based spatiotemporal sampling design. After specifying a suitable model, the accuracy and uncertainty of the estimation and prediction, using either a likelihood-based (frequentist) approach or a Bayesian approach, are intricately linked to the selection of the sampling design (Senarathne et al., 2023). The sampling design serves as the foundation upon which the entire process is built, influencing the representativeness of the data and, consequently, the reliability of the parameter estimation and subsequent predictions (Mateu and Müller, 2013).

## 4 Selection of spatiotemporal sampling design

A sampling method or mechanism is needed to select a subset of spatial and temporal units to obtain the sampling design $S=\left\{\left(u_{1},t_{1}\right),\ldots ,\left(u_{n},t_{n}\right)\right\}$. Relative to space and time, the selection methods can be classified as:

• Separate selection: sampling locations and times are chosen independently from each other.
• Conditional selection: first, sampling times (or locations) are selected, and then, for each chosen time (or location), the sampling locations (or times) are subsequently chosen.
• Joint selection: sampling locations and times are selected concurrently.

The objective of the study and practical limitations determine the selection method to adopt. Consider for example a study focusing on variations in a vector population across both spatial units (e.g. villages) and temporal units (e.g. months). In separate selection, a set of locations $S_{1}=\left\{u_{1},\ldots ,u_{n_{1}}\right\}$ is initially chosen, followed by selecting a set of time points $S_{2}=\left\{t_{1},\ldots ,t_{n_{2}}\right\}$, regardless of the temporal characteristics of each location; therefore the sampling design is $S=S_{1}\times S_{2}=\left\{\left(u,t\right):u\in S_{1},t\in S_{2}\right\}$. In conditional selection, after selecting the sampling locations $S_{1}=\left\{u_{1},\ldots ,u_{n_{1}}\right\}$, the selection of sampling times $S_{2}\left(u\right)=\left\{\left(u,t_{1}\right),\ldots ,\left(u,t_{n_{2}\left(u\right)}\right)\right\}$ for each location $u\in S_{1}$ are determined based on specific conditions, such as seasonal variations. Consequently, the sampling times at one location may differ from those at another location and the sampling design is given by $S=\underset{u\in S_{1}}{⋃}S_{2}\left(u\right)$. Finally, in joint selection, both sampling locations and times $\left(u_{1},t_{1}\right),\ldots ,\left(u_{n},t_{n}\right)$ are simultaneously chosen based on the common factors affecting the spatial and temporal variation. In presence of correlation between location and time, conditional (weak-medium correlation) or joint (strong correlation) selection should be preferred to separate selection.

In any of these cases, units can be selected by various combinations of random or non-random methods, preferential or non-preferential sampling, and adaptive or non-adaptive techniques.

## 5 Selection of units

### 5.1 Random and deterministic sampling

In a random or probability sampling, units are randomly selected such that the probability of each unit being included in the sample is known. Random sampling designs are often used to eliminate potential subjective biases in the selection of units. Common random sampling methods are summarized in Table 2. Conversely, in non-random or deterministic sampling, units are selected based on criteria such as geometry (grid sampling and coverage or infill sampling), convenience (opting for accessible sites, such as those in urban areas or along roads and favorable times), specific targeting (choosing locations or times suspected of high disease rates), or even arbitrary selection without a particular objective in mind (Brus, 2022). Several theoretical investigations and empirical evidence suggest that random sampling designs are reasonably more efficient for parameter estimation than non-random designs, while, provided that the model parameters are known, (non-random) regular grid sampling designs generally lead to more efficient prediction than random designs (Brus, 2022). To capture the advantages of both random and regular grid samplings, random inhibitory sampling designs, which require any pair of sample locations and/or times to be separated by at least a specified inhibition threshold but not constrained to regularity, can be adopted (Chipeta et al., 2017).

**Table 2:** Common random sampling methods and their assumptions about variability of the study variable.

Sampling method	Selection mechanism	Assumptions
Simple random sampling	Each unit has an equal probability of being selected for sampling.	All units are easily accessible, the study variable is relatively homogeneous across units.
Systematic random sampling	The first unit is selected randomly, and the rest of the units are selected following a specific regular pattern.	The study variable exhibits a regular or predictable pattern.
Stratified random sampling	First units are divided into strata (subgroups) based on some characteristic or properties of the variable of interest. Then, simple random sampling or systematic sampling is used within each stratum.	The study variable has a relatively homogenous variation within each stratum, although variation can significantly differ between strata.
Cluster random sampling	First units are organized into clusters, and then a specific subset of these clusters is chosen either randomly or systematically.	The study variable has high variability between clusters, but homogeneous and low variability within individual clusters.

### 5.2 Preferential sampling

Preferential sampling can be classified as (i) convenience sampling, and (ii) purposive sampling. In the first case, units are selected for inclusion in the sample for convenience (e.g. ease of access and/or safety for the observers). In the second case, units satisfying certain criteria are selected from the statistical population under study. Preferential sampling can be non-probabilistic or probabilistic.

In preferential or informative sampling, the selection of units depends on either the study variable or both the study variable and the factors that affect it (Pennino et al., 2019). Preferential sampling may be used to target data collection on a specific season or time period, as well as habitats or ecosystems of interest. It is the practical solution for conducting surveillance for a rare disease or species given the low probability to detect a positive sample in many areas. In addition, with limited resources for data collection, preferential sampling allows for prioritization of data collection in specific regions and times where the study variable is expected to exhibit significant variability or trends (Aubry et al., 2024). Preferential sampling designs are efficient when there is reliable knowledge about the distribution of the study variable and it is clear how to account for the introduced bias and misrepresentation; otherwise, non-preferential sampling designs should be preferred (Conn et al., 2017). In particular, in model-based inference, it is crucial to account for the effects of preferential sampling on parameter estimation and prediction. Ignoring the biases introduced by preferential sampling can result in misleading inferences, compromising the accuracy and reliability of the results (Diggle et al., 2010).

### 5.3 Adaptive sampling

Adaptive sampling is a probabilistic preferential sampling where new units are added sequentially to an already existing sampling design (Seber and Thompson, 1996). Selection of the next units is conditional to the data obtained from previously selected units in the sampling process. Since information gained from the existing sampling units guides the selection of new units, it is known as a form of preferential sampling with similar advantages and limitations. Adaptive sampling designs are effective for clustered or rare populations, when resources (time, budget, or personnel) for data collection are limited, and the study variable exhibits significant spatial or temporal variability. In contrast, non-adaptive sampling designs are often sufficient when the study variable remains homogeneous and predictable (Seber and Salehi, 2012).

Vector populations often exhibit dynamic spatial and temporal heterogeneities, with clusters of higher activity or density in certain areas and during specific seasons. Adaptive sampling designs are highly beneficial in resource-limited settings where ongoing surveillance of vector populations is essential. These designs facilitate spatial mapping, enabling the identification of critical areas where interventions can achieve the greatest health impact (Chipeta et al., 2016). Moreover, if the priority is collecting the largest possible number of mosquitoes (i.e. for genetic analyses), adaptive sampling can allow for an informed allocation of sampling effort to the areas and times with the highest densities; or can be used to target intervention such as vector control measures on the areas with largest vector index (Monteiro et al., 2024).

## 6 Which sampling method to use?

The selection of a sampling method is based on the general principles outlined above and summarized in Fig. 2. For instance, a common strategy is to select spatial and temporal units independently using a non-random, non-adaptive, and often preferential (targeted) method for sampling in time, and a random stratified and adaptive method for sampling in space. In this case, stratification of spatial units can be aided by the use of climate and environmental data (Sedda et al., 2019).

Another example is to first select temporal units using a non-random, non-adaptive, and non-preferential method, such as a regular grid, and then at each sampling time perform a random systematic, non-adaptive, and preferential sampling method for selecting spatial units based on the knowledge that the vectors are known to cluster regularly, as for example along a linear feature, such as riverbanks.

However, sampling designs can be selected based on optimization of a cost or loss or utility function. In fact, a loss function, denoted as L(S), can be employed to evaluate the effectiveness of any sampling design S. Then, the most appropriate sampling design can be chosen by minimizing (maximizing) this loss (utility) function. Minimizing the loss function can aid the definition of the optimal number of samples needed to achieve the study objectives, as well as their locations and times of survey. Depending on the objective(s) of study, the definition of the loss function is commonly based on the accuracy of the estimates produced by the sampling design, the level of uncertainty in the model’s parameters, the improving spatial/temporal coverage, the financial cost associated with implementing the sampling design, or a combination of two or more of these or other factors (Müller, 2007). Thus, quantities such as estimation variance, prediction error, financial and logistical costs, and penalties for violating sampling constraints such as minimum distance between spatial or temporal units can be used to define a suitable loss function. To achieve an optimal sampling design, all sampling considerations and study objectives must be quantified and integrated into a unified mathematical expression for the loss function L(S). For example, in the absence of any prior knowledge about the study variable, and if sampling costs are the same for all spatial and temporal units, the sample size n is specified based on the available budget or logistic that maximize the accuracy of the spatiotemporal prediction of the study variable. In this context maximizing spatial and temporal coverage for the sampling design minimizes the mean shortest distance loss function (Brus, 2022):

$$L\left(S\right)=\frac{1}{Nm}\sum_{i=1}^{N}\sum_{j=1}^{m}\left(\min_{\left(u,t\right)\in S}\left|\left({u_{i},t}_{j}\right)-\left(u,t\right)\right|\right)$$

If prediction is the primary aim and prior knowledge about the study variable is available (adaptive design), the loss function can be defined based on prediction performance criteria such as the mean square prediction error and prediction variance (Andrade-Pacheco et al., 2020), the Kullback-Leibler divergence distance or the entropy criterion (Senarathne et al., 2023). For instance, suppose no assumptions are made about the sampling costs and distribution of the study variable Y(u,t), and a prior estimate denoted by $\tilde{Y}\left(u,t\right)$ is available across all spatial and temporal units, then the mean square prediction error:

$$L\left(S\right)=\frac{1}{Nm}\sum_{i=1}^{N}\sum_{j=1}^{m}{\left(\hat{y}\left(u_{i},t_{j}\right)-\tilde{y}\left(u_{i},t_{j}\right)\right)}^{2}$$

can be minimized to obtain the optimal sampling, where $\hat{Y}\left(u,t\right)$ denotes the predicted value for the study variable based on the sample S.

On the other hand, if the primary objective is to estimate model parameters, the formulation of the loss function can be based on the variance of the estimated parameters or the Fisher information matrix of the model (Müller, 2007). For example, suppose the study variable Y(u,t) follows a Gaussian (normal) distribution and there are constant sampling costs across all spatial and temporal units, the loss function L(S) can be defined as the negative determinant of the Fisher information matrix of the random vector consisting of Y(u,t) at the sampling units (u,t)∈S. Then, minimizing L(S) results in an optimal model-based sampling design for estimation of the trend (expected value) of Y(u,t).

Note that in an adaptive sampling design, insights gained from initial data collections and analyses can be used to refine the loss function and sampling strategy as more information becomes available.

## 7 General guidance for spatiotemporal surveillance of vectors

This general guidance aims to set some common principles on the potential spatiotemporal sampling designs to be employed in vector surveillance. It is therefore necessarily broad and does not consider the numerous possibilities and field scenarios disease-vector managers can encounter in their work; and it does not provide guidance on the optimal number of surveillance sites, target vector indexes, seasonality, and quality of the available data (van de Straat et al., 2021). In addition, the following guidance does not distinguish between passive and active surveillance. For example, in Europe, passive surveillance is mostly implemented in the context of mosquito and tick vectors, while active surveillance is commonly implemented country-wide for mosquitoes and biting midges, whereas the active surveillance of ticks and sand flies most often focuses on selected parts of the countries (ECDPC, 2021). Most of the active vector surveillance is classified as ‘limited in time’ and this holds for all vector groups.

Basic principles on how a surveillance framework should be designed are provided by the main animal and human health organizations. In fact, in the WHO Global Vector Control Response strategy (2017–2030) (WHO, 2017) one of the nine priority activities is the ‘National vector surveillance systems strengthened and integrated with health information systems to guide vector control’. The strategy goes further and defines the pillars of actions to achieve effective vector control. Pillar 3 entitled ‘Enhancing vector surveillance and monitoring and evaluation of interventions’ by recognizing that ‘*the capacity of vectors to transmit pathogens and their susceptibility to vector control measures can vary by species, location and time, depending on local environmental factors*’ (page 28), identify the following good practices.

• Vector surveillance should be routinely conducted at representative sites in areas where vector-borne diseases are endemic as well as those with low or no ongoing transmission but receptivity to pathogen transmission.
• Surveillance information must enable stratification of areas for further investigation or prioritized resources.
• Programmes must be aware of the entomological and vector-borne disease situation in neighboring countries and more broadly in the region as well as globally. Data repositories and sharing have an essential role in this.
• Data from outside of the health sector should also be utilized. These data include information on urban planning, housing, water and sanitation as well as from the agricultural sector such as insecticide usage. In addition to climate and environmental conditions, these data can be used in public health policy and planning (e.g. to predict changes in vector populations or the risk of disease transmission).
• Geographical information system techniques and technologies should be leveraged to aid data interpretation.

Stratification as defined by the WHO (WHO, 2012) is the classification of disease endemic areas by their epidemiological and ecological characteristics, and it is recognized as mandatory tool to prioritize allocation of resources. For example, the WHO Global Malaria Programme adopts stratification to differentiate provinces or districts according to four levels of malaria endemicity: with 100, 1–100, < 1 and 0 cases per 1000 population per year. No further guidance is provided on how should look like a surveillance spatial design, but WHO stresses that a direct link should be established between vector surveillance and vector control so that the results of surveillance are implemented in vector control interventions.

The Animal Health Organisation (WOAH, 2023) in its recommendations on surveillance for arthropod vectors of animal diseases, provides guidance on the design of the sampling plan for the risk of vector introduction in a new area. In its three-stage hierarchy plan the stages are: (i) stratification based on ecological criteria (where possible) assessing the risk of introduction; (ii) subdivision of strata into spatial sampling units; and (iii) within each sampling unit the establishment of actual sampling sites. However, if adequate entomological, epidemiological and historical data and/or expert opinion exists, the sampling plan may be refined or targeted by defining strata which are as homogeneous as possible with respect to known or suspected risk-factors. In addition, the Animal Health Organisation recommends that the number and size of the spatial sampling units should be defined to provide appropriate estimates of the indicators used, however not providing further information on which approach should be used. They also suggest that more intensive sampling might be carried out in strata where vector presence seems most likely, based on biological or statistical criteria, clearly leaning towards a preferential sampling.

Given the described health organizations’ surveillance recommendations, and considering the advantages and disadvantages of each of the explored sampling designs, the following guidelines are provided.

(i) *For pilot studies with limited sampling effort* (for example just a few sampling locations), any sampling design can be used under the consideration of the caveats affecting each of them (Case et al., 2024).
(ii) *For pilot studies with good sampling effort*, coverage (Case et al., 2024) should be prioritized by spreading locations in space and time to provide accurate representation of the study area and time period. Within a design-based inference approach, statistical methods such as the nearest neighbor and spatial interpolation analyses are commonly used to ensure accurate and unbiased predictions.
(iii) *In the absence of entomological information*, but with the availability of climate and/or environmental and/or epidemiological data, a stratified lattice with close pairs or stratified inhibitory design should be considered in space (Diggle and Lophaven, 2006; Sedda et al., 2019), while the temporal repetition should be based on the assumed or expected seasonality, for which power tests could be employed (Sedda et al., 2023). These sampling designs balance optimal coverage for prediction with the inclusion of closely spaced random pairs for accurate parameter estimation.
(iv) *When entomological information is available*, along with other climate, environmental, and/or epidemiological data, a probabilistic adaptive sampling framework should be used (Fairley et al., 2022), unless the number of samples is very large – for which a mixture of random and lattice approaches could provide better optimization towards different targets (Conn et al., 2017; Andrade-Pacheco et al., 2020; Case et al., 2022). In case the criterion aims to improve the reliability of prediction maps, the maximum rather than the mean reduction in uncertainty represents an “all-in” approach (Case et al., 2024) – as for example minimization of prediction variance as opposed to entropy (Andrade-Pacheco et al., 2020).
(v) *Avoid using non-probabilistic preferential sampling*, unless in pilot studies with limited sampling effort, since it produces a statistically biased estimation of the trends, patterns, and variability of the process under study (Conroy et al., 2023). In fact, it has been shown that the bias in the mean increases with the covariance between the propensity and the variable of interest, such as species richness, abundance, etc. (Aubry et al., 2024). Sometimes is impractical to fully apply a probabilistic sampling; however, it is often possible to consider probability sampling structures (e.g. stratification, cluster sampling, sampling with probability proportional to an importance factor) for at least some part of the sampling process, as for example when targeting breeding or resting sites (Nusser et al., 2008).

As stressed above, and independently from the approach (model-based or design-based), any prior information about the spatial and temporal heterogeneity of the study variable should be utilized to create strata for stratified sampling. In adaptive sampling, prediction maps based on previous data, along with estimates of accuracy, costs, and sampling restrictions, can be used to develop a loss function. This function helps determine the optimal sampling design for subsequent stages.

Several tools and software packages provide robust frameworks and methods for designing (Pantalone et al., 2022; Dumelle et al., 2023) and implementing spatial and spatiotemporal sampling designs for vector-borne disease surveillance and across other fields such as environmental monitoring. For the latter an example is MSANOS, a software application that employs spatial model-based and design-based, adaptive and non-adaptive optimization methods and objective functions, to redesign monitoring networks (Barca et al., 2015). This shows that tools from different subjects can be useful for the optimization of vector surveillance programmes.

## 8 Conclusions

Spatiotemporal sampling designs play a crucial role in mapping and understanding vectors and their transmitted pathogens, providing valuable insights into the patterns and trends of diseases over space and time. Specifying a spatiotemporal sampling design should be the first step in any entomological and/or epidemiological study before data collection. A well-defined spatiotemporal sampling design minimizes biases that could distort the results and ensures that the data collected are representative of the vector population. In addition, it avoids the costs associated with over-sampling or the risks of under-sampling. By focusing efforts on high-priority areas and times identified by probabilistic spatiotemporal sampling, limited financial resources are used more efficiently, and the collected data provide the most valuable information for understanding the spatial and temporal patterns of the study variable. This in turn can lead to more robust and insightful conclusions such as early detection of trends and patterns, enabling timely potential interventions. In conclusion, the weaker the assumptions are made about the process, the better option is the use of a random sampling design. On the contrary, when strong assumptions can be made, probabilistic preferential and adaptive sampling will return faster and better gains. All these methods can enhance the accuracy and efficiency of vector surveillance, aiding in effective resource allocation and the development of disease prevention strategies.

## Funding

This work was funded by the 10.13039/100000865Bill and Melinda Gates Foundation under the UCSF Malaria Elimination Initiative project ‘Equipping countries for evidence-based malaria intervention strategies’. Abdollah Jalilian and Luigi Sedda are also supported by the 10.13039/100010269Wellcome Trust 10.13039/501100000272NIHR - 10.13039/100004440Wellcome Partnership for Global Health Research Collaborative Award, CEASE (220870/Z/20/Z). The funder had no role in study design and analysis, decision to publish, or preparation of the manuscript.

## Ethical approval

Not applicable.

## CRediT authorship contribution statement

**Abdollah Jalilian:** Conceptualization, Formal analysis, Investigation, Methodology, Writing – original draft, Writing – review & editing, Project administration. **Jorge Mateu:** Investigation, Methodology, Writing – review & editing. **Luigi Sedda:** Conceptualization, Investigation, Methodology, Writing – review & editing, Funding acquisition, Project administration.

## Data availability

The data supporting the conclusions of this article are included within the article.

## Declaration of competing interests

The authors declare that they have no known competing financial interests or personal relationships that could have appeared to influence the work reported in this paper.
